# The organum vasculosum of the lamina terminalis and subfornical organ: regulation of thirst

**DOI:** 10.3389/fnins.2023.1223836

**Published:** 2023-09-04

**Authors:** Jiaxu Wang, Fenglin Lv, Wei Yin, Zhanpeng Gao, Hongyu Liu, Zhen Wang, Jinhao Sun

**Affiliations:** ^1^Department of Anatomy and Neurobiology, School of Medicine, Shandong University, Jinan, Shandong, China; ^2^Institute of Sport and Exercise Medicine, North University of China, Taiyuan, China; ^3^Department of Cardiology, Affiliated Hospital of Shandong University of Traditional Chinese Medicine, Jinan, China

**Keywords:** organum vasculosum of the lamina terminalis, subfornical organ, thirst, renal regulation, circadian regulation

## Abstract

Thirst and water intake are regulated by the organum vasculosum of the lamina terminalis (OVLT) and subfornical organ (SFO), located around the anteroventral third ventricle, which plays a critical role in sensing dynamic changes in sodium and water balance in body fluids. Meanwhile, neural circuits involved in thirst regulation and intracellular mechanisms underlying the osmosensitive function of OVLT and SFO are reviewed. Having specific Na_x_ channels in the glial cells and other channels (such as TRPV1 and TRPV4), the OVLT and SFO detect the increased Na^+^ concentration or hyperosmolality to orchestrate osmotic stimuli to the insular and cingulate cortex to evoke thirst. Meanwhile, the osmotic stimuli are relayed to the supraoptic nucleus (SON) and paraventricular nucleus of the hypothalamus (PVN) via direct neural projections or the median preoptic nucleus (MnPO) to promote the secretion of vasopressin which plays a vital role in the regulation of body fluid homeostasis. Importantly, the vital role of OVLT in sleep-arousal regulation is discussed, where vasopressin is proposed as the mediator in the regulation when OVLT senses osmotic stimuli.

## Introduction

In mammals, thirst-induced water intake is regulated by the circumventricular organs (CVOs), including the organum vasculosum of the lamina terminalis (OVLT) and subfornical organ (SFO), which are essential sites for monitoring blood osmolality, sensing multiple hormone stimuli, and influencing water intake via regulating thirst (Seay et al., 2020). They are located near the ventricular space in the midline of the brain and are a special structure located in the third and fourth ventricles of the brain (Sisó et al., 2010). The OVLT and SFO are important in sensing Na^+^ in body fluids, regulating plasma osmolality, and maintaining fluid homeostasis (Gizowski and Bourque, 2018). They are highly vascularized, which enables them to participate in communication between the brain and peripheral tissues through sensation and secretion (Kiecker, 2018). Lacking the blood–brain barrier, the OVLT and SFO can quickly monitor changes in the concentration of Na^+^ in body fluids, where Na_x_ channels on the surface of glial cells play an important role in sensing blood osmolality.

The OVLT was described as the supraoptic crest early. Brizzee and Wislocki performed earlier investigations into the cell structure of the supraoptic crest in cats and rats, respectively (Wislocki and Leduc, 1952; Brizzee, 1954). Until 1976, the ultrastructure of OVLT was studied using Japanese quail (Mikami, 1976). After the 1990s, the neuroanatomical study of OVLT began to emerge gradually. Several studies have indicated that the OVLT has extensive nerve connections with the SFO and median preoptic nucleus (MnPO), which play a critical role in controlling vasopressin (VP) release. Meanwhile, it has been shown that OVLT GABAergic neurons project to the MnPO to modulate the release of noradrenaline (Ushigome et al., 2004). Moreover, the OVLT projects to the suprachiasmatic nucleus (SCN), which has been proven to participate in osmolality homeostasis, secretion of VP, and circadian regulation. Since then, OVLT has been shown to exert multiple homeostatic functions from fluid homeostasis and thermoregulation to cardiovascular regulation; additionally, it may be involved in the regulation of circadian rhythms.

The SFO has been identified as the structure of the laminae terminalis (Fry and Ferguson, 2021), and it is also involved in the regulation of osmotic pressure and thirst (Noda and Hiyama, 2015). By immunohistochemistry, Na_x_ channels were found to be on the SFO, which is the structural basis for the SFO to monitor changes in cerebrospinal fluid osmolality (Noda and Hiyama, 2005). On the basis of perceived osmolality changes, SFO can induce thirst production or reduce salt intake through neural pathways such as GABAergic (Noda and Hiyama, 2015; Abbott et al., 2016), and ANG II can also induce thirst production and water intake by acting on SFO (Fitzsimons, 1969; McKinley et al., 1998). Additionally, the MnPO is involved in the regulation of osmotic pressure and thirst by receiving projections from the OVLT and SFO (McKinley et al., 2015).

There is an excellent paper concerning the regulation of body fluid homeostasis, which is focused on the Na^+^ sensors, thirst, and the mechanisms underlying (Noda and Matsuda, 2022). In this review, thirst-evoking and osmotic regulation by the OVLT and SFO and mechanisms of OVLT and SFO sensing osmotic stimuli are explored. Furthermore, we focus on the neural circuits involved in the regulation of body fluid homeostasis and a vital hormone involved in fluid homeostasis and circadian regulation and come up with an intriguing idea about the role of OVLT in sleep regulation.

## Thirst and water intake

### Thirst evoked by the OVLT and SFO

The first report proposing a possibly specific role of OVLT in thirst was obtained in rats, showing that prolonged thirst provoked the emergence of LHRH-containing fibers in the OVLT (Krisch, 1978). Meanwhile, it was shown that SFO lesions also attenuated thirst and water intake (Buggy and Fisher, 1976). Subsequently, another study revealed that central or peripheral administration of angiotensin II-induced thirst by acting on periventricular tissue of the anteroventral third ventricle. Meanwhile, bilaterally electrolytic lesions of tissue surrounding the anteroventral third ventricle (AV3V) abolished angiotensin II induced drinking response (Buggy and Johnson, 1978). The following experiment on dogs shows that lesions of OVLT only significantly reduce osmotically-induced drinking with intravenous infusion of hypertonic saline (Thrasher et al., 1982). As well, rats with ablation of OVLT by an electrode drink markedly more excessive water than rats with intact OVLT even though they are not treated with hypertonic saline. Meanwhile, without treatment of hypertonic saline, rats with lesions of both the OVLT and SFO show higher desires for water and salt than that with OVLT lesion single, indicating the synergy between the OVLT and SFO in thirst regulation (Fitts et al., 2004).

The OVLT and SFO, as brain regions for monitoring cerebrospinal fluid osmotic pressure, play an important role in regulating plasma sodium concentration and thirst. Intracerebroventricular (ICV) infusions of hypertonic saline altered the intensity and the distribution pattern of c-fos expression neurons in OVLT of rats (Solano-Flores et al., 1993). As revealed recently, individual OVLT neuron is sufficient to sense the elevated levels of NaCl and Ang II (Kinsman et al., 2020), and OVLT neurons expressing the angiotensin 1A receptor are activated by increased plasma osmolarity and then robustly triggers thirst (Leib et al., 2017).

Meanwhile, the immunohistochemical technique ensures the crucial role of the OVLT in the regulation of blood osmolality in rats, in which c-fos expression and TRPV1 protein expression levels in the neurons of the OVLT ascend in response to angiotensin II-induced hypertension, indicating that OVLT neurons are activated when exposed to high blood osmolality (Issa et al., 2012). As revealed recently, individual OVLT neuron is sufficient to sense the elevated levels of NaCl and Ang II and then contributes to thirst and water intake (Kinsman et al., 2020). Under conditions of water deprivation, the GABAergic neurons with Na_x_ channels of the SFO can sense the increase in sodium concentration in cerebrospinal fluid, thus activating GABAergic interneurons to inhibit the activity of neurons conveying osmotic stimuli to subsequent nuclei, while under conditions of sodium depletion, thirst-driving neurons are inhibited via a CCK-mediated pathway, thus increasing the concentration of Na^+^ in body fluids (Matsuda et al., 2020). The SFO is a key structure for the human body to control salt intake, and if the genes that code Na_x_ channel proteins are knocked out in mice, abnormal salt intake will occur. Under dehydrated conditions, wild-type mice reduce salt ingestion, but the knock-out mice ingest excessive salt (Hiyama et al., 2004). Na_x_ channels, which are present in the SFO but also on the surface of the OVLT, stimulate the cellular processes of astrocytes and ependymal cells to regulate sodium homeostasis of body fluids when they are activated by Na^+^ (Noda and Hiyama, 2015).

The mechanisms of various types of thirst have been summarized in studies. Thirst caused by a decrease in extracellular fluid volume is first detected by pressure receptors that detect arterial pressure due to its reduced volume and transmits signals to regions such as the nucleus tractus solitarius and ventrolateral medulla (Badoer et al., 1992; Zardetto-Smith et al., 1993; Thunhorst et al., 1998; McKinley and Johnson, 2004). Examples testifying to this idea are that in hypovolemic states, SFO, MnPO, and OVLT all have associated elevated c-fos expression (Potts et al., 2000; Gizowski and Bourque, 2018). At the same time, we have previously described the effect of the hypertonic state on sleep; in fact, sleep also triggers an increase in osmolality producing thirst, which is due to the prolonged absence of water intake during sleep, while respiration and urine production still excrete water. This can be alleviated in humans by anticipating thirst, and it has been shown that mice drink significantly more water 2 h before sleep (Gizowski et al., 2016), a condition associated with the SCN vasopressinergic neurons and the OVLT, and that neural projections from vasopressin neurons of the SCN to OVLT are necessary for rhythmic thirst before sleep (Abrahamson and Moore, 2001; Gizowski et al., 2016).

In conclusion, all of the above mechanisms of thirst production are inseparable from OVLT or SFO, validating the important role of OVLT and SFO in the production of thirst.

### Neural circuits

**Figure 1:**
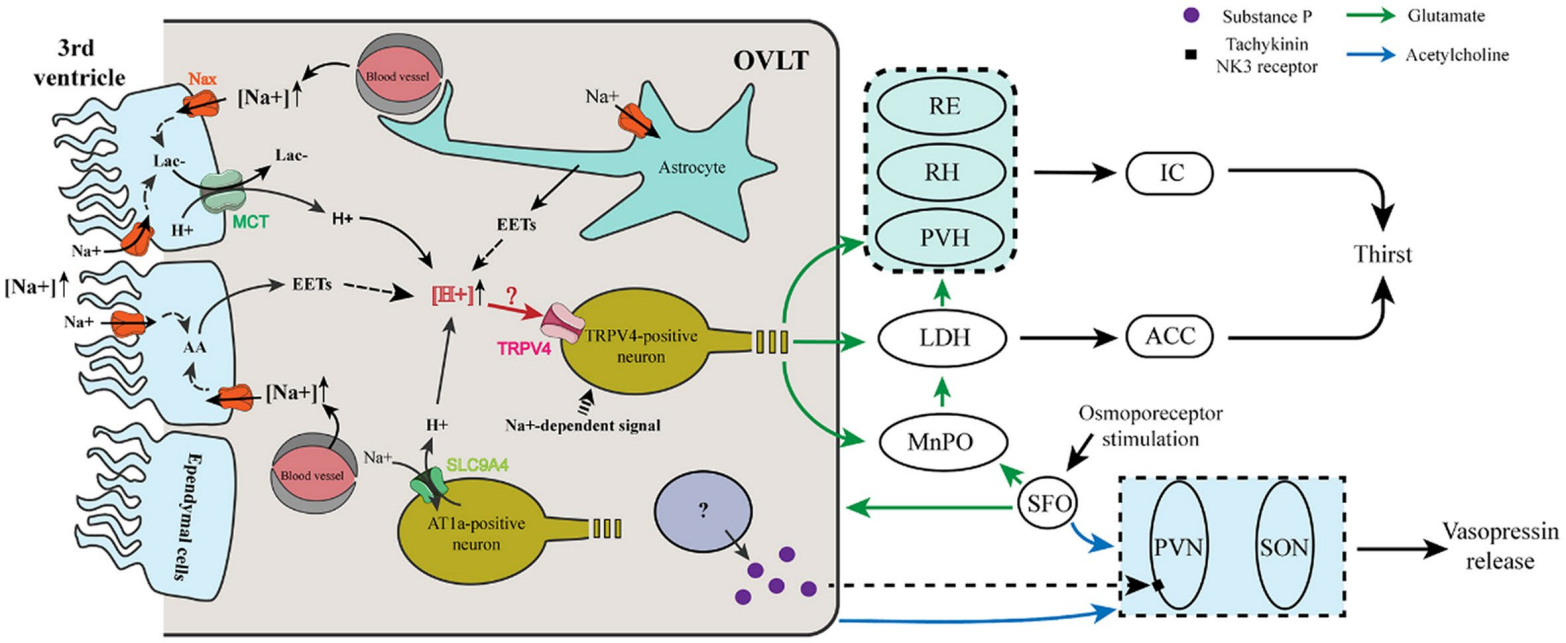
Cellular mechanism and neural pathway involved in the thirst and vasopressin regulation of OVLT and SFO. (i) Ependymal cells and astrocytes sense Na^+^ in the third ventricle and blood vessels via Na_x_ channels to produce lactate and EETs that enhance the extracellular [H^+^]. Meanwhile, AT1a-positive neurons sense Na^+^ from blood vessels via a Na^+^ sensor (SLC9A4), which imports extracellular Na^+^ and then exports H^+^. Increased extracellular H^+^ acts on TRPV4-positive neurons to evoke glutamatergic thirst-promoting efferents to the PVH, RH, and RE, which relay the signals to the IC, and to the MnPO, and LDH, which relays the signals to the ACC. The LDH also receives glutamatergic projections from the MnPO. (ii) After sensing osmotic stimuli, the OVLT and SFO cholinergic projections to the SON and PVN enhance their action of vasopressin release. Meanwhile, a population of OVLT neurons may release substance P to promote the release of vasopressin from the PVN. The SFO innervates the OVLT and MnPO via glutamatergic projections.

Regarding the neural pathway involved in thirst regulation, except for the OVLT, many brain regions, such as the MnPO, SFO, and dorsal thalamus, participated in thirst regulation (Figure 1). Both the OVLT and SFO have been revealed as key sites in the brain for sensing the sodium level of body fluid, with specialized Na_x_ channels and integrating multiple circulating fluid balance signals with receptors for Ang II and glucose (Noda, 2006; Morita et al., 2016; Paes-Leme et al., 2018; Izumisawa et al., 2019; Kinsman et al., 2020). Current evidence shows that the osmotic signal is relayed to the insular and cingulate cortex from the OVLT via the thalamus. Immunohistochemical analysis, CTb staining, and Fos staining ascertained the trajectory of osmotic signals from the OVLT to the insular and anterior cingulate cortex. Furthermore, projections from the OVLT to the paraventricular nuclei of the thalamus (PVH), rhomboid nuclei (RH), and reuniens nuclei (RE) of the thalamus are relayed to the insular cortex. Projections from the OVLT to the medial part of the laterodorsal thalamic nuclei (LDH) are relayed to the cingulate cortex. The majority of thalamic regions projecting to the insular and cingulate cortex are along the thalamic midline (Hollis et al., 2008).

A previous study showed that the SFO projects densely to the OVLT and MnPO, whereas the OVLT and MnPO have much denser projections to the thalamus than to the SFO, and glutamatergic MnPO ^Adcyap1^ neurons have been reported to project broadly to the PVH, the medial part of laterodorsal thalamic nuclei. Importantly, each pathway is sufficient to evoke thirst and drinking (Leib et al., 2017). Intriguingly, *in situ* hybridization histochemistry and immunohistochemistry show that glutamatergic and GABAergic neurons coexist in the same nucleus of the SFO, OVLT, and MnPO, indicating that their excitatory or inhibitory function depends on which population is activated and where they project to (Grob et al., 2003). Correspondingly, voracious water intake occurs in mice when glutamatergic neurons are optogenetically stimulated in the OVLT, while reduced water consumption occurs in mice when GABAergic neurons are optogenetically stimulated in the OVLT (Abbott et al., 2016), indicating that glutamate or GABA is used to evoke or inhibit thirst by the OVLT.

Moreover, it has been reported that osmosensitive OVLT neurons project to osmosensitive MnPO neurons to drive MnPO neuron activation to project to magnocellular neurons of the thalamus in response to hyperosmotic stimulation (Honda, 2003). Given the present clues, when exposed to plasma hyperosmolarity, we propose that OVLT glutamatergic neurons project to the paraventricular, rhomboid, and reuniens nucleus of the thalamus, whose projections innervate the insular cortex; OVLT glutamatergic neurons project to the medial part of laterodorsal thalamic nuclei, whose projections innervate the cingulate cortex; SFO glutamatergic projections innervate OVLT glutamatergic neurons that project to the insular and cingulate cortex; MnPO integrates glutamatergic projections from OVLT and SFO and projects to magnocellular neurons of PVH and LH; every single pathway could evoke thirst and water intake.

### Mechanisms of OVLT and SFO sensing osmotic stimuli

Mechanisms of OVLT and SFO sensing osmotic stimuli are presented in Figure 1. Na_x_ channels in the OVLT and SFO have been demonstrated to be involved in the regulation of thirst. Na_x_ channels existing in the glial cells (ependymal cells and astrocytes) of the OVLT and SFO functioned as sensors for Na^+^, and a high level of Na^+^ promoted the synthesis of epoxyeicosatrienoic acids (EETs) from arachidonic acid (AA), which acted on transient receptor potential vanilloid 4 (TRPV4) within the OVLT and then enhanced water intake (Sakuta et al., 2016, 2020b). Regarding the correlation between Na_x_ channel activation and synthesis of EETs, elevated extracellular [Na^+^] activated Na^+^/K^+^-ATPase via Na_x_ channels in glial cells and then enhanced their metabolic activities to produce more lactic acid (Shimizu et al., 2007). Meanwhile, lactate is also released by glial cells of the OVLT via monocarboxylate transporters (MCTs) (Nomura et al., 2019).

Another Na^+^ sensor (SLC9A4) exists in the OVLT and SFO expressed in AT1a-positive neurons sensing extracellular [Na^+^]. Additionally, it can promote water intake by acting as a sodium (Na^+^)/hydrogen (H^+^) exchanger when the extracellular [Na^+^] increases and then induces the activation of acid-sensing channel 1a (ASIC1a) (Sakuta et al., 2020a). However, H^+^ produced by Na_x_-positive glial cells is involved in blood pressure regulation by acting on ASIC1a (Nomura et al., 2019). Thus, the former experiment may eliminate the receptor involved in the regulation of thirst and blood pressure. We propose that both of the Na^+^ channels enhance the extracellular [H^+^] within the OVLT and SFO and may transmit the signal by activating TRPV4-positive neurons to evoke thirst.

Additionally, OVLT and SFO neurons can be activated by nonionic-induced hypertonicity, such as mannitol. When the extracellular osmolarity increases to cause a decrease in the cell volume, microtubules in neurons provide a pushing force to drive transient receptor potential vanilloid 1 (TRPV1) activation utilizing a uniquely interweaved scaffold interacting with the C-terminus of TRPV1 and ultimately induce the depolarization of the osmosensory neurons (Mannari et al., 2013; Prager-Khoutorsky et al., 2014; Sakuta et al., 2016). It has been revealed that TRPV1 mediates the osmoreception effect of OVLT via nonselective ion channels permeable to K^+^ and Na^+^ (Ciura and Bourque, 2006).

Taken together, osmosensory neurons of the OVLT and SFO detect both Na^+^ concentrations and osmolarity to regulate fluid homeostasis, while how OVLT and SFO neurons sense hypovolaemic stimuli needs to be further elucidated. Moreover, water-channel aquaporin 4, which may contribute to cell deformation to trigger the activation of osmosensory neurons, is found within the OVLT except for the middle triangular area (Kálmán et al., 2019). However, the underlying mechanisms have not been explained.

## Vasopressin regulation

### The regulation of vasopressin secretion

The supraoptic nucleus of the hypothalamus (SON) as well as the suprachiasmatic nucleus (SCN) and magnocellular neurons of the paraventricular nucleus of the hypothalamus (PVN) secretes vasopressin, which could be stimulated when the OVLT and SFO sense the osmotic stimuli (Benarroch et al., 2006; Jansen et al., 2019; Li et al., 2021). Rather than the SCN, vasopressin secreted by the SON and PVN seems to be involved in the osmoregulation. Hyperosmolality can activate osmosensitive channels, triggering downstream signal transmission to maintain fluid homeostasis (Liu et al., 2018; Levi et al., 2021). The initial studies gave out the original clues of the secretion regulation of the OVLT on vasopressin. After knife cuts were placed between the level of the OVLT and SON, rats showed enhanced water consumption and urine volume which were abolished by bilateral nephrectomy, indicating a crucial role of the neural pathway between the OVLT and SON in fluid regulation (Bealer et al., 1984). The more exact evidence for the vasopressin secretion regulated by the OVLT was obtained from the dog, in which electrolytic ablation of the OVLT significantly decreased plasma arginine vasopressin and reduced the magnitude of the drinking and vasopressin increase in response to hypertonic saline (Thrasher and Keil, 1987).

The regulation of SFO on osmotic pressure is also closely related to vasopressin, there is evidence that electrical stimulation of SFO can increase the release of vasopressin in the posterior pituitary (Ferguson and Kasting, 1986). The injection of relaxin and hypertonic saline can activate the external area of SFO, and the activated nerve cells project axons to SON, PVN, and MnPO (McKinley et al., 1998). Moreover, ANGII sensitive SFO neurons may enhance the excitability of vasopressin-secreting neurons in PVN to respond to circulating ANGII (Tanaka et al., 1987). Electrophysiology shows that the SFO-SON pathway is involved in the release of vasopressin in response to SFO angiotensin II stimulation (Johnson et al., 1999).

In recent years, more advanced technology has brought insights into the correlation between OVLT and SFO and vasopressin secretion. An experiment in c-fos-mRFP1 transgenic rats shows that osmotic stimulation (hypertonic saline administration) induces the activation of the OVLT, SFO, and MnPO as well as raises vasopressin-enhanced green fluorescence in the SON and PVN, suggesting that the osmosensitive brain regions may innervate the SON and PVN (Fujihara et al., 2009). Fos-immunohistochemistry and tract-tracing techniques show that osmosensitive OVLT and SFO neurons project to the PVN and SON, as well as innervate the MnPO. Moreover, using Fos-immunohistochemistry and tract-tracing techniques, it is revealed that the MnPO integrates osmolality stimuli from the OVLT and SFO and conveys them to vasopressin-containing neurons in the SON and PVN via direct neural innervations (Oldfield et al., 1991; Larsen and Mikkelsen, 1995; Shi et al., 2008; Bichet, 2018). Patch-clamp recordings were obtained in SON neurons with the stimulation of the OVLT. However, results show that the OVLT can only activate vasopressin-containing neurons in the SON (Yang et al., 1994). Moreover, the excitatory postsynaptic potentials (EPSPs) of magnocellular neurosecretory cells in the SON are enhanced when the OVLT is exposed to hypertonic saline, which is inhibited in the presence of the gamma-aminobutyric acid (GABA) (Richard and Bourque, 1995).

Horseradish peroxidase iontophoresis into the PVN nucleus has labeled neurons within the OVLT and SFO projecting to the PVN (Silverman et al., 1981). Indeed, Fos and CTB immunostaining shows that hyperosmolality stimuli specifically activate the OVLT neurons that project to the PVN monosynaptically and hypertonic saline with ascending gradient increased Fos-positive OVLT neurons gradually, indicating that PVN-projecting OVLT neurons participate in the neuroendocrine of the PVN responses to hyperosmolality (Shi et al., 2008). Patch-clamp recordings and retrograde tract-tracing show that labeled PVN-projecting neurons within the SFO are osmosensitive and sensitive to angiotensin II. Meanwhile, a transient potassium conductance dominates a unique electrophysiological profile of these labeled neurons, whose mechanism remains to be clarified (Anderson et al., 2001).

### Neural innervations and cellular mechanisms

As to the mediator participating in the process, intensive cholinergic fibers are located in the SFO, OVLT, and vasopressin-secretion nuclei (SON and PVN) (Xu et al., 2001a). ICV injections of carbachol, a cholinergic receptor agonist, enhance the activity of a significant number of SON-projecting neurons within the OVLT, SFO, and MnPO and promote water intake as well as the release of vasopressin (Xu et al., 2001b), indicating that the cholinergic pathway plays an important role in the osmoregulation of vasopressin.

As to the neural circuits (Figure 1), the MnPO innervates with the OVLT and SFO reciprocally and projects intensely to the SON, lesser to the PVN. Meanwhile, an electron microscope found that the MnPO efferent fibers formed synapses with vasopressin-containing neurons (Oldfield et al., 1991). In a later observation using the electron microscope, biotinylated dextran was used as a tracer to trace labeled terminals anterogradely and results showed that fibers from the MnPO roughly formed a monosynaptic connection with vasopressin neurons (Armstrong et al., 1996). The MnPO possesses dense NMDA receptors and NMDA infusion into the AV3V significantly raises the plasma vasopressin, which is abolished by pretreatment with an NMDA receptor antagonist, MK-801 (Yamaguchi and Watanabe, 2002; Schwartz et al., 2008). Furthermore, AV3V injection of GABA receptor antagonist induces a marked increase of plasma vasopressin, and a GABA receptor agonist induces the decrease of plasma vasopressin in contrast (Yamaguchi and Yamada, 2008), indicating that the GABA acted as the inhibiting mediator in the secretion of vasopressin.

One experiment marked thirst-triggering neurons in the SFO with the transcription factor ETV-1 and optogenetic activation of them trigger thirst. Moreover, the OVLT and MnPO are observed to receive projections from the ETV1 neuronal population. Furthermore, the neurons projecting to the SON and PVN are glutamatergic (Oka et al., 2015). The aforementioned clues show that there exist two types of neurons with respective functions in the vasopressin secretion in osmoregulation. One is osmotic neurons sensing and conveying osmotic stimuli mentioned above, and another one is cholinergic/ GABAergic neurons participating in the secretion of vasopressin. However, the mechanism or the mediator participating in the process remains mysterious.

Moreover, it is shown that the Tachykinin NK_3_ receptor in the magnocellular neurons of the PVN plays a critical role in vasopressin secretion in response to the osmotic challenge, in which hyperosmolarity induced by hypertonic saline can activate the Tachykinin NK_3_ receptor to enhance the secretion of vasopressin (Haley and Flynn, 2007). Immunohistochemical detection has verified that the osmosensitive organs, especially the OVLT and SFO contained substance P (Brownstein et al., 1976; Spitznagel et al., 2001), an agonist of the Tachykinin NK_3_ receptor. That indicates that substance P may act as a neurotransmitter of the neural projections from the OVLT and SFO to the PVN to regulate vasopressin secretion when they sense the osmotic stimuli.

## Renal regulation

It was reported that rats with knife cuts posterior to the OVLT excreted significantly less sodium than the control under the condition of water deprivation (Bealer et al., 1983). And the same effect was seen in dogs with OVLT lesions (Ramsay and Thrasher, 1984), indicating the important role of the OVLT in the regulation of natriuresis. Also, an early study showed that rats with electrolytic AV3V lesions showed a reduction of natriuresis compared with the sham, while activation of the SFO with cholinergic agonist showed an increase of natriuresis compared with the sham, also indicating that the SFO could regulate the natriuresis (Colombari et al., 1992). Therefore, the OVLT and SFO could exert effects on the regulation of blood osmotic balance via regulating renal excretion.

The sympathetic nerve activity (SNA) plays a vital role in the body homeostasis, for example, cardiovascular regulation (Colombari et al., 1992). Furthermore, renal sympathetic nerve activity (RSNA) plays a vital role in body fluid homeostasis (Malpas and Burgess, 2000). The OVLT, as well as the SFO and MnPO, have been shown to be crucial components participating in blood pressure regulation. Lesions of the OVLT, SFO, and MnPO separately all attenuated the pressor response to Ang II or excessive NaCl (Starbuck and Fitts, 1998; Vieira et al., 2010; Ployngam et al., 2012). Intravenous infusion of angiotensin II markedly increases c-fos expression in the OVLT and angiotensin II responsive cells in the OVLT were recorded in hypothalamic brain slices, indicating that angiotensin II directly activates neurons within the OVLT (Knowles and Phillips, 1980; McKinley et al., 1992; Oldfield et al., 1994; Sunn et al., 2003). Similar to the OVLT, the SFO is the main brain region where ANG II affects cardiovascular function, and electrophysiological recordings have shown that ANG II injection or plasma hypernatremia increases the firing rate of half of the SFO neurons and that electrical stimulation of the SFO leads to an increase in blood pressure via sympathetic pathways (Ferguson and Renaud, 1984; Gutman et al., 1988). It was also shown that mice with spontaneous hypertension had higher firing rates of SFO neurons projecting to the PVN and higher excitability to ANG II than normal mice (Miyakubo et al., 2002).

**Figure 2:**
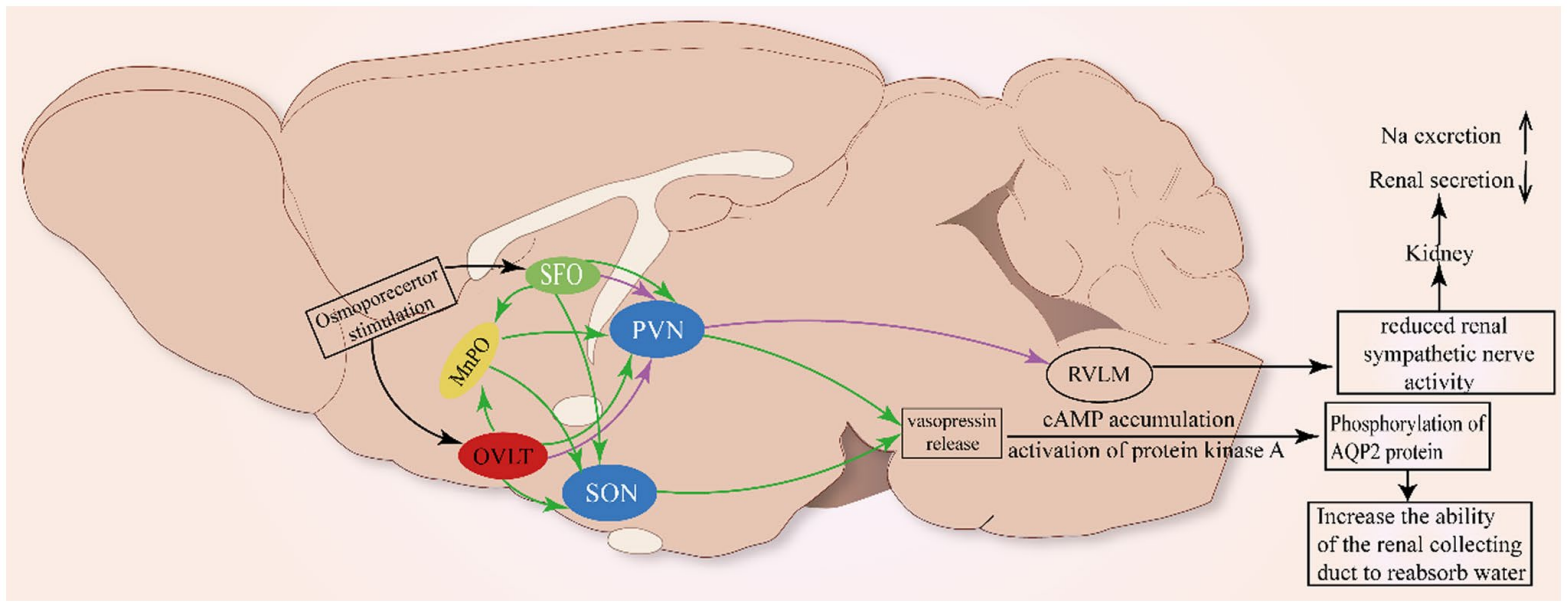
Neural pathway involved in the renal regulation of OVLT and SFO. As shown in Figure 1, glia cells (ependymal cells and astrocytes) in the OVLT sense Na^+^ from the third ventricle and blood vessels via Na_x_ channels to produce EETs and lactate that enhance extracellular [H^+^]. The MnPO integrates projections from the OVLT and SFO to innervate the SON and PVN to promote the secretion of vasopressin to act on the kidney to regulate natriuresis. Additionally, the OVLT and SFO project directly to the PVN to inhibit renal sympathetic nerve activity and renal secretion.

The latest study indicates that optogenetic stimulation of angiotensin type1a receptor-positive neurons in the OVLT could activate magnocellular neurons within the SON and PVN, facilitating vasopressin secretion, which is adequate to evoke a blood pressure increase, and that effect can be attenuated by vasopressin-1a-receptor antagonists (Frazier et al., 2021). Apart from Ang II, plasma Na^+^ has been considered a crucial pressor effector for decades. Kengo Nomura identified Na_x_ channels that detect the increase in plasma [Na^+^] expressed in glial cells in the OVLT, and Na_x_-positive glial cells export H^+^ to activate OVLT neurons projecting to the PVN via ASIC1a, which is responsible for enhancing the SNA (Nomura et al., 2019). The neural circuits involved are shown in Figure 2.

Meanwhile, ANG II-induced reactive oxygen species (ROS) can be involved in the development of neurogenic hypertension through the SFO-PVN-RVLM pathway (Braga et al., 2011). The above mechanism has been clearly described in studies where ANG II acts on AT1 receptors to activate ROS receptors, which then alters calcium homeostasis and activates the neural pathway between the SFO and PVN (Anderson et al., 2001; Zimmerman et al., 2005; Peterson et al., 2006), thereby promoting the release of vasopressin. Meanwhile, optogenetic stimulation of OVLT neurons markedly activates the rostral ventrolateral medulla (RVLM) bulbospinal neurons to orchestrate sympathetic signals and significantly elevated renal, splanchnic, and lumbar sympathetic nerve activity (Stocker et al., 2022), indicating that the OVLT-RVLM pathway is involved in blood pressure regulation. Retrograde labeling has marked PVN neurons projecting to the RVLM (Nomura et al., 2019). The above clues indicate that the PVN acts as a site to integrate projections from the OVLT, SFO, and MnPO to the RVLM to influence the activity of SNA.

Some studies have shown that central administration of angiotensin II can inhibit renin secretion in rats and renal sympathetic nerve activity in sheep, reflecting the role of forebrain structures in renal regulation (Weekley, 1992; May and McAllen, 1997). The reverse infection caused by the injection of the pseudorabies virus into the kidney indicated that the neural nuclei such as SFO and OVLT did have neural projections with the kidney (Sly et al., 1999). Meanwhile, it is reported that reduced RSNA could enhance sodium excretion (Karaaslan et al., 2005). Simultaneously, vasopressin also contributes to the maintenance of normal osmotic pressure by acting on the kidney. The effect of vasopressin on the kidney is typically to activate the V2 receptor, which is highly expressed in the collecting ducts of rats, mice, and humans, to reabsorb water (Phillips et al., 1988). The V2 receptor is associated with the cAMP accumulation and activation of protein kinase A, which in turn phosphorylates the AQP2 protein and cAMP reactive binding protein (CREB) (Gonzalez et al., 2020). The phosphorylation of AQP2 results in the translocation of AQP2 to the plasma membrane of the top cell in the main cell of the collecting duct, which increases the reabsorption of water (Sands et al., 1987). Finally, the plasma osmotic pressure is reduced and the urine osmotic pressure is increased.

At the same time, vasopressin can promote the release of renin from the renal collecting duct, causing the activation of the renin-angiotensin-aldosterone system (RAAS), which together with water retention causes an increase in blood pressure (Gonzalez et al., 2016). There is evidence that increased brain sodium concentration can reduce the RSNA to promote urinary sodium loss (May et al., 2010), and rapid infusion of hypertonic saline reduces renal SNA, which has been found in rabbits (Weiss et al., 1996; Badoer et al., 2003). In humans, long-term high-salt diets can lead to elevated SNA and a rise in RSNA can lead to an increase sodium retention (Winternitz et al., 1980).

## Thirst and circadian regulation

### Circadian thirst

During sleep, the body gradually becomes dehydrated as it breathes and sweat evaporates without replenishing water, mammals can alleviate this effect by improving the circadian rhythm of osmotic pressure regulation (Gizowski and Bourque, 2018). Recent studies have found that rodents’ water intake significantly increases before bedtime, with the amount of water consumed in the 2 h before bedtime being greater than in the previous 2 h (Gizowski et al., 2016). This rhythmic thirst before sleep has been found to be related to VP (Gizowski et al., 2017). The level of cerebrospinal fluid VP began to rise sharply in the hours before the end of the active phase and peaked in the first half of the inactive phase (Gizowski et al., 2017). To alleviate the effects of dehydration during sleep, SCN VP neurons become active 2 h before sleep in mice, and activate the OVLT by releasing VP to act on postsynaptic V1a receptors and downstream non-selective channels (Gizowski et al., 2016), which induces rhythmic thirst in mice before bedtime.

### Clock time regulated by the OVLT

**Figure 3:**
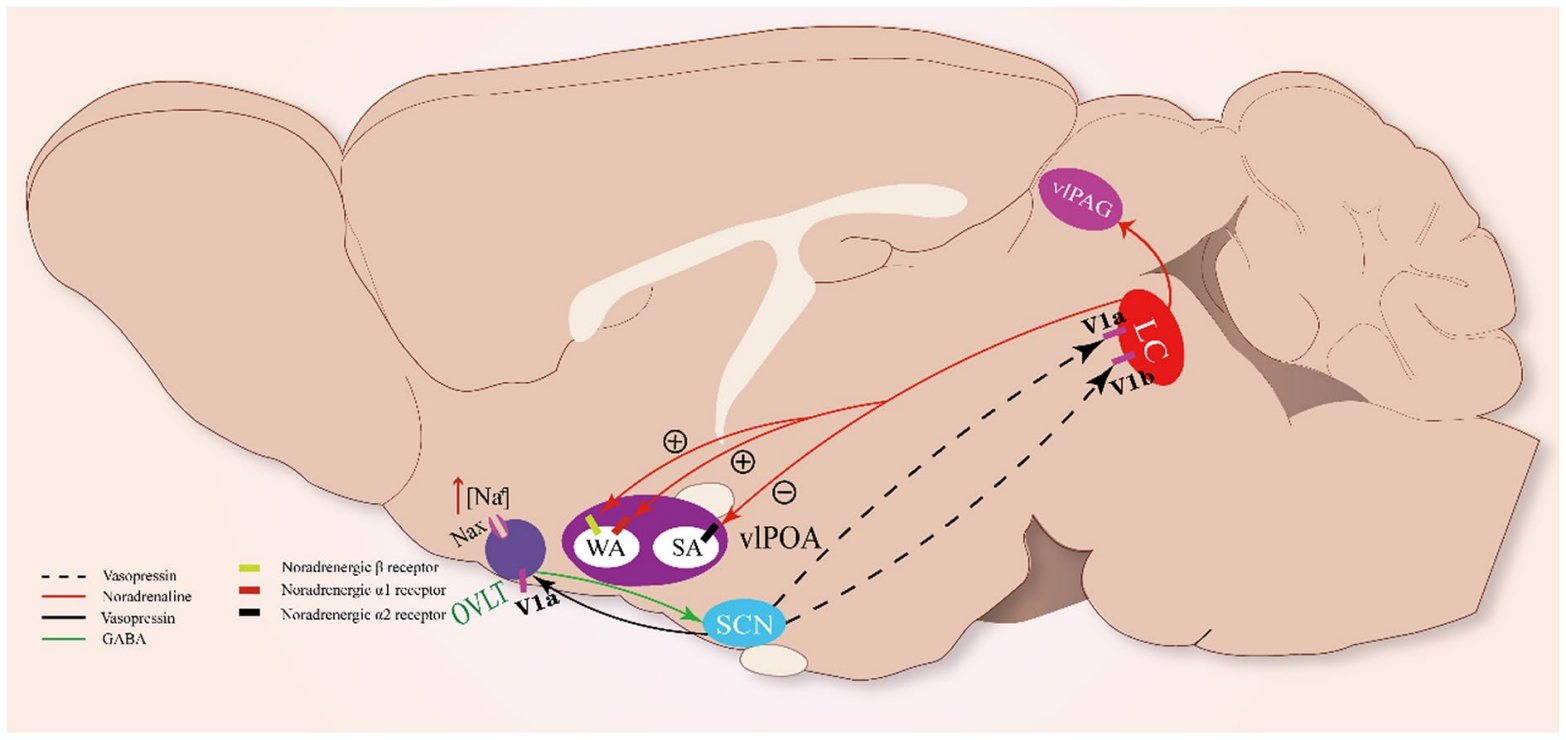
Sleep-arousal regulation of the OVLT and SCN. When the OVLT senses osmotic stimuli, it promotes VP secretion from the SCN via GABA. VP promotes the activity of LC NAergic neurons via V1a and V1b receptors. LC NAergic neurons inhibit vlPOA sleep-active neurons to promote sleep via noradrenergic α_2_ receptors and activate wake-active neurons to promote wakefulness via noradrenergic β- and α₁-receptors. Meanwhile, LC NAergic neurons activate vlPAG astrocytes via noradrenergic α₁-receptors.

It has been revealed that the OVLT could relay hypertonic saline-induced hyperosmolality signals to VP neurons of the SCN to enhance the release of VP via excitatory projections from GABAergic neurons and phase-advanced the clock time of mice, demonstrating that the OVLT can drive clock neurons within the SCN to carry out unscheduled homeostatic responses (Gizowski and Bourque, 2020) (Figure 3). The above clues show that the OVLT can regulate arousal action via the regulation of VP secretion. However, how the arousal action is regulated by VP remains to be further explained.

In rats, vasopressin-immunoreactive cells were observed in the locus coeruleus (LC), and VP induced excitatory effects on it (Caffé and van Leeuwen, 1983; Olpe et al., 1987). The LC neurons were found to possess vasopressin 1a (V1a) and 1b (V1b) receptors through which VP could significantly promote the excitability of LC noradrenergic neurons (Campos-Lira et al., 2018). The LC has been regarded as one of the essential modulating centers of circadian rhythm (Aston-Jones and Bloom, 1981; Schwarz and Luo, 2015). Meanwhile, it has been revealed that noradrenergic LC neurons participate in the regulation of sleep–wake rhythm (González and Aston-Jones, 2006). Optogenetic stimulation of noradrenergic LC neurons causes REM sleep-to-wake transitions, while optogenetic inhibition of these neurons significantly induces wake-to-NREM transitions (Carter et al., 2010), indicating that noradrenergic LC neurons are essential to maintain the normal duration of the sleep–wake cycle. Thus, OVLT may regulate the circadian rhythm mainly by orchestrating the secretion rhythm of the VP that acts on the LC.

Noradrenergic LC neurons could impose wakefulness-promoting effects on a cluster of brain structures with noradrenergic β- and α₁-receptors, such as the MnPO, LH, dorsal raphe, and vlPAG. Projections from the LC enhance the alertness of these structures via norepinephrine (NE) (Samuels and Szabadi, 2008; Berridge et al., 2012). Recently, two main neural pathways involving the vlPOA and vlPAG underlying the arousal-promoting action of the LC have been revealed: the LC-vlPOA and LC-vlPAG neural pathways. Noradrenergic LC neurons project directly to the vlPOA and mediate rapid arousal by inhibiting sleep-active neurons and activating wake-active neurons. NE exerts opposite actions on sleep-active neurons and wake-active neurons of the vlPOA via noradrenergic α_2_ receptors and noradrenergic β- and α₁-receptors, respectively (Liang et al., 2021). Meanwhile, noradrenergic LC neurons innervate vlPAG astrocytes via noradrenergic α₁-receptors to promote arousal (Porter-Stransky et al., 2019).

In summary, current evidence shows that the OVLT contributes to the phase-advanced clock time when sensing osmotic stimuli. Meanwhile, the LC plays a vital role in this shift of clock time via innervations to the vlPOA and vlPAG. However, further evidence supporting this idea is needed.

## Conclusion

In summary, thirst regulation needs vital osmotic sensors, OVLT and SFO, and innervations among different nuclei. Meanwhile, the role of the OVLT in sleep-arousal regulation is emphasized and explored. With specific Na_x_ channels, hyperosmolality of the third ventricular or blood could be detected by the OVLT and SFO, and the osmotic signals orchestrated by the OVLT and SFO are relayed to the cingulate cortex and insular cortex to regulate thirst via the MnPO, paraventricular, rhomboid, and reuniens nuclei of the thalamus. Vasopressin plays an important role in the regulation of blood osmolality, and the OVLT and SFO could regulate renal excretion by regulating the secretion of VP and regulating RSNA via the PVN-RVLM pathway. Furthermore, after sensing the osmotic change, the OVLT could orchestrate the osmotic stimuli into the sleep–wake oscillation by regulating VP secretion from the SCN. Noradrenergic LC neurons serve as the integrating site, changing the osmotic stimuli to sleep–wake oscillation by relaying the signal to the vlPOA and vlPAG to phase-advance clock time. This review shows the critical role of the OVLT and SFO in the regulation of thirst and fluid homeostasis, emphasizing that the OVLT and SFO have emerged as the major sensor to regulate thirst and body fluid homeostasis.

## Author contributions

All authors listed have made a substantial, direct, and intellectual contribution to the work and approved it for publication.

## Funding

This work was supported by the National Science Foundation of China. The project reference was 81871044.

## Conflict of interest

The authors declare that the research was conducted in the absence of any commercial or financial relationships that could be construed as a potential conflict of interest.

## Publisher’s note

All claims expressed in this article are solely those of the authors and do not necessarily represent those of their affiliated organizations, or those of the publisher, the editors and the reviewers. Any product that may be evaluated in this article, or claim that may be made by its manufacturer, is not guaranteed or endorsed by the publisher.
